# Typhoid and paratyphoid fever: a call to action

**DOI:** 10.1097/QCO.0000000000000479

**Published:** 2018-08-17

**Authors:** Malick M. Gibani, Carl Britto, Andrew J. Pollard

**Affiliations:** Oxford Vaccine Group, Department of Paediatrics, University of Oxford, and NIHR Oxford Biomedical Research Centre, Oxford, UK

**Keywords:** antimicrobial resistance, diagnostics, paratyphoid fever, typhoid conjugate vaccines, typhoid fever

## Abstract

**Purpose of review:**

Enteric fever remains a major global-health concern, estimated to be responsible for between 11.9 and 26.9 million cases annually. Long-term prevention of enteric fever will require improved access to safe drinking water combined with investment in sanitation and hygiene interventions. In the short-to-medium term, new control strategies for typhoid fever have arrived in the form of typhoid Vi-conjugate vaccines (TCVs), offering hope that disease control can be achieved in the near future.

**Recent findings:**

The diagnosis of enteric fever is complicated by its nonspecific clinical presentation, coupled with the low sensitivity of commonly used diagnostics. Investment in diagnostics has the potential to improve management, to refine estimates of disease burden and to facilitate vaccine impact studies. A new generation of reliable, diagnostic tests is needed that are simultaneously accessible, cost-effective, sensitive, and specific. The emergence and global dissemination of multidrug-resistant, fluoroquinolone-resistant, and extensively drug-resistant (XDR) strains of *Salmonella Typhi* emphasizes the importance of continued surveillance and appropriate antibiotic stewardship, integrated into a global strategy to address antimicrobial resistance (AMR). Current empirical treatment guidelines are out of date and should be updated to respond to local trends in AMR, so as to guide treatment choices in the absence of robust diagnostics and laboratory facilities. In September 2017, the WHO Strategic Advisory Group of Experts (SAGE) immunization recommended the programmatic use of TCVs in high burden countries. Ongoing and future studies should aim to study the impact of these vaccines in a diverse range of setting and to support the deployment of TCVs in high-burden countries.

**Summary:**

The advent of new generation TCVs offers us a practical and affordable public-health tool that – for the first time – can be integrated into routine childhood immunization programmes. In this review, we advocate for the deployment of TCVs in line with WHO recommendations, to improve child health and limit the spread of antibiotic-resistant *S. Typhi*.

## BACKGROUND

Enteric fever remains a major public health problem, affecting millions of people every year and disproportionately impacting low- and middle-income countries. The global enteric fever landscape has transformed steadily over the past two decades, illustrated by the emergence and dissemination of multidrug-resistant (MDR) and fluoroquinolone-resistant strains of *Salmonella Typhi*, an increasing burden of *S. Paratyphi* A infection in South Asia and the development of a new generation of typhoid conjugate vaccines (TCVs). The recent approval and impending deployment of TCVs is a cause for optimism in efforts to achieve control of enteric fever globally. Nevertheless, several challenges remain. This review aims to summarize the contemporary enteric fever landscape, focussing on burden of disease, diagnosis, treatment, and prevention of enteric fever. In addition to summarizing several new research advances, we aim to identify knowledge gaps that could be addressed in future studies. 

**Box 1:**
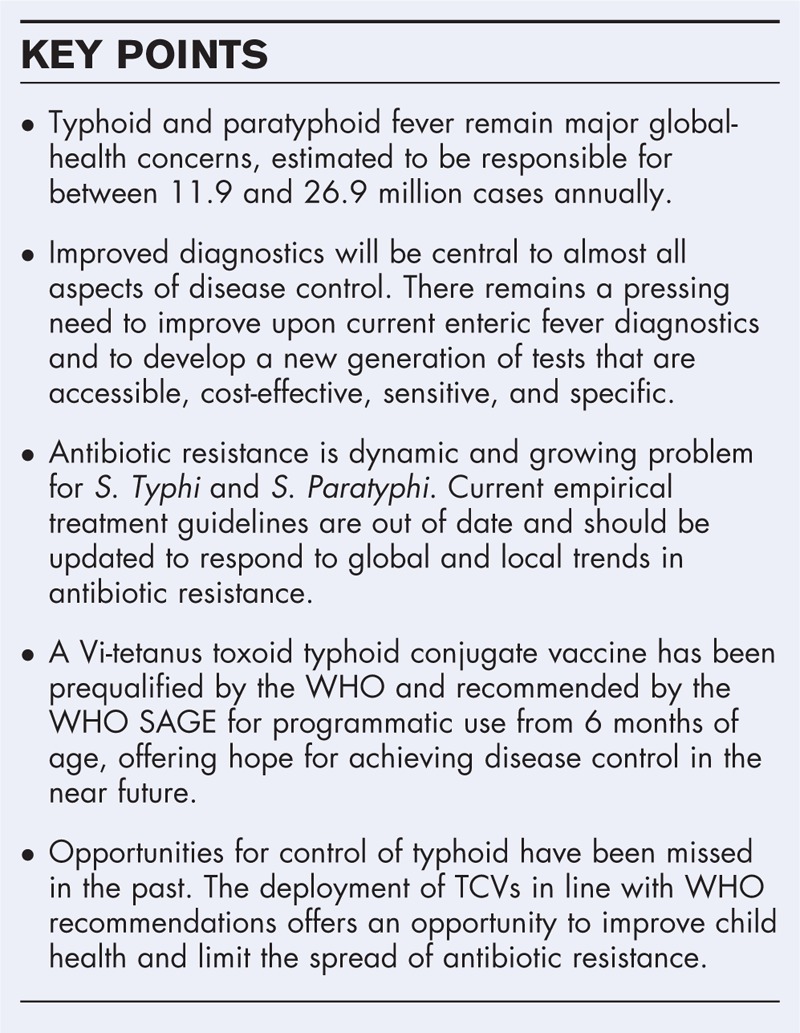
no caption available

## THE DISEASE AND PATHOGENESIS

Typhoid fever is caused by infection with *S. enterica* subspecies *enterica* serovar typhi (*S. Typhi*), a Gram-negative facultative anaerobic bacillus. Paratyphoid fever results from infection with the related organism *S. enterica* subspecies *enterica* serovar paratyphi (*S. Paratyphi*), which is divided into three subtypes – *S. Paratyphi* A, B, and C. *S. Typhi* and *Paratyphi* are collectively referred to as typhoidal *Salmonella* serovars and infection with either can result in the clinical syndrome of enteric fever. Unlike other *S. enterica* serovars, *S. Typhi,* and *Paratyphi* are human-restricted pathogens that cause a systemic illness progressing to an asymptomatic chronic carrier state in some individuals.

Transmission of *S. Typhi* and *Paratyphi* occurs through consumption of contaminated food or water via short-cycle or long-cycle transmission. Short-cycle transmission is defined as the contamination of food and water in the immediate environment through inadequate hygiene and sanitation measures, either by shedding from acute or chronic carriers. Long-cycle transmission is defined as contamination of the broader environment, such as pollution of water supplies by sewage, or inadequate treatment of piped water. The relative contribution of each transmission mode may vary depending on the epidemiological context and may differ between *S. Typhi* and *Paratyphi*[1].

The clinical presentation of typhoid fever is highly variable, ranging from a mild-illness characterized by low-grade fever and malaise, through to a severe life-threatening systemic illness with multiple complications, including intestinal perforation, intestinal hemorrhage, and encephalopathy [2]. Ingestion of bacteria and systemic invasion is followed by a short-lived period of asymptomatic primary bacteraemia. The incubation period, typically lasts 7–14 days, but can range from 3 to 60 days dependent, in part, on the size of the inoculum. Symptoms are usually nonspecific and include fever, malaise, anorexia, headache, arthralgia, myalgia, nausea, abdominal discomfort, and dry cough. Sporadic, asymptomatic, shedding of the bacteria in the stool can occur prior to the development of symptomatic disease. Clinical signs may include high fever, relative bradycardia, abdominal tenderness, hepatomegaly, splenomegaly, or rose-spots. *S. Paratyphi* may cause a milder disease than *S. Typhi*[3^▪^], although field data from the largest case series to-date, comprising 609 enteric fever patients in Nepal suggest that both serovars cause an indistinguishable clinical syndrome [4].

In the absence of effective antimicrobial therapy, approximately 1–5% of patients with acute typhoid infection are thought to become chronic carriers. Risk factors for chronic carriage include the presence of gallstones, female sex, older age, and inadequate treatment courses. Chronic carriers may be responsible for maintaining low-level transmission of disease and thus could complicate disease eradication efforts through sanitation and vaccination programs [5]. Accurate identification and treatment of chronic carriers will likely form an important component of future disease control efforts.

The pathogenesis of enteric fever and host response to infection are reviewed by Dougan and Baker [6]. A key virulence factor expressed by most strains of *S*. *Typhi* is a polysaccharide capsule, termed the Vi (virulence) antigen. The Vi-capsule is encoded by the *viaB* locus, which comprises several genes required for biosynthesis and export of the capsule. In the absence of the Vi-capsule, *S. Typhi* is inherently more sensitive to killing in serum than other serovars, such as *S. Typhimurium*[7]. The Vi-capsule possesses immunomodulatory properties that are thought to contribute to disease pathogenesis, including limiting complement deposition, reducing immune activation, assisting with phagocytosis evasion, and inhibiting serum bactericidal activity [7,8]. The Vi capsule forms the principal component of parenteral typhoid vaccines, including new conjugate vaccines. Vi-antigen is expressed by other bacteria including *Citrobacter freundii*, *S. Paratyphi* C, and *S. dublin*.

Advances in genomics studies offer insights into the pathogenic mechanisms of *S. Typhi* and *Paratyphi*. The genome of *S. Typhi* is notable for the accumulation of multiple pseudogenes, thought to reflect the host-restriction properties of typhoidal *Salmonella* as a similar process has been observed in other host-restricted pathogens [9]. In addition, *S. Typhi* possess approximately 300–400 specific genes not found in other *S.* serovars. Many of these gene products are encoded on *Salmonella* pathogenicity islands relatively unique to *S. Typhi* (e.g., SPI-5, SPI-15, SPI-17, and SPI-18) [6]. For example, *S. Typhi* and *Paratyphi* A possess a recently described exotoxin termed the typhoid-toxin, which is postulated to have a central role in pathogenesis of enteric fever (reviewed in ref. [10]). The characterization of virulence factors that may have an important role in disease pathogenesis could aid the development of novel vaccines for typhoidal *Salmonella*.

## BURDEN OF DISEASE

Over recent years, several groups have published studies refining estimates of the global burden of enteric fever, which have presented additional surveillance data from sub-Saharan Africa and improved our understanding of the disease epidemiology by modelling for specific risk factors [11^▪▪^,^12^,13^▪^]. Estimates for the annual burden of disease range from 11.9 to 26.7 million cases, with 128 00 to 216 500 deaths. The global burden of enteric fever is concentrated in low- and middle-income countries. The disease has been essentially eliminated as a public-health problem in high-income countries over the past century, owing to improvements in water quality, sanitation, and hygiene [14].

A consistent finding of burden-of-disease studies performed to date is the high incidence of typhoid fever in South and South-East Asia. Within these regions, the epidemiology of typhoid is complicated by marked inter- and intracountry variation. For example, data from the Diseases of Most Impoverished program have described incidence rates varying from 24.2/100 000 in Vietnam to 493.5/100 000 in parts of India. Recent studies have demonstrated high rates of typhoid fever in rural areas of Cambodia and West Africa, suggesting that the disease is not restricted to urban settings with poor sanitation systems [11^▪▪^,^15^].

The heterogeneity of typhoid disease epidemiology may be more pronounced in Africa. Surveillance performed in two sites in Kenya between 2006 and 2009 found that the incidence of blood-culture proven typhoid fever in rural and urban sites varied from 29 up to 247-cases/100 000 person-years [16]. Recent data from the Typhoid Fever Surveillance in Africa Program highlighted marked differences in incidence rates between sites in Africa with adjusted rates ranging from 0 in Sudan to 383/100 000 person years in Burkina Faso. This study also demonstrated marked intracountry variation, with higher rates in rural Ghana compared with urban settings [11^▪▪^].

Data from ongoing surveillance studies suggest that an estimated 27% of typhoid fever cases requiring medical attention occur in children aged 0–4 years, of which a substantial proportion (∼30%) occur at ages below 2 years [17]. These data are supported by a recent meta-analysis, underlining the large burden of disease in preschool children [18]. Age-specific incidence may vary by country, risk factors, and force of infection.

Ongoing surveillance studies (including the Surveillance of Enteric Fever in Asia Project, Severe Typhoid in Africa Program, and the Strategic Typhoid Alliance across Africa programs aim to better characterize the burden of severe typhoid disease, refine our understanding of age distribution, and to better characterise the role of chronic carriers in transmission dynamics. It is hoped that data generated from these studies will help to inform future prevention strategies [19,20].

### Paratyphoid fever

The proportion of disease caused by *S*. *Paratyphi*, as compared with *S. Typhi*, is highly variable depending on the geographic context. *S. Paratyphi* is thought to be responsible for approximately one-fifth of all enteric fever cases [21]. Increasing incidence of disease caused by *S*. *Paratyphi* A has been reported over the past 2 decades such that this serovar is responsible for an increasing proportion of enteric in parts of Asia, including in Nepal [22^▪^], Cambodia, [23] and China [24]. For example, in a recent retrospective study *S. Paratyphi* A was responsible for 86% of enteric fever cases in Phnom Penh, Cambodia between 2013 and 2015, increasing from 26% in the period 2008–2012 [23]. The highest burden of paratyphoid fever is estimated to occur in China, with an estimated annual incidence of 150 cases/100 000 person-years. The available data for Africa indicate that *S*. *Paratyphi* are responsible for less than 2% of enteric fever cases [24].

There is currently no available vaccine against *S*. *Paratyphi* A. The Ty21a vaccine may confer some cross protection against *S*. *Paratyphi* B, estimated at up to 49% [25]. Individuals vaccinated with Ty21a have detectable cross-reactive humoral immune responses against *S. Paratyphi* A *in vitro*[26–28]. However, field studies in highly endemic areas have shown no conclusive effect of Ty21a on the burden of *S. Paratyphi* A [29]. Several candidate paratyphoid vaccines are in development, including live attenuated vaccines and lipopolysaccharide conjugate vaccines. The increasing global incidence of paratyphoid fever would make a bivalent vaccine, providing protection against both *S. Typhi* and *Paratyphi*, a valuable public health tool [30].

## DIAGNOSIS

There remains a pressing need to improve upon current enteric fever diagnostics and to develop a new generation of tests that are accessible, cost-effective, sensitive, and specific [31]. Bone marrow culture is considered the ‘gold-standard’ diagnostic test for enteric fever, but frequently impractical to perform in many endemic settings. Blood culture is the mainstay of typhoid and paratyphoid diagnosis. A recent systematic review estimated the average diagnostic sensitivity of blood culture to be 61.1% [95% confidence interval (CI) 51.9–70.3%] [12,32].

Rapid diagnostic tests (RDTs) for typhoid and paratyphoid fever could theoretically be combined with clinical algorithms to differentiate febrile patients to guide management, particularly in areas lacking well-equipped laboratory facilities. Several RDTs for enter fever diagnosis have been developed, the most commonly of which are the Typhidot/Typhidot-M test, the TUBEX test and Test-It Typhoid. The current generation of typhoid RDTs has only modest sensitivity and specificity determined in meta-analyses and there is insufficient evidence to support their exclusive use for the diagnosis and management of enteric fever [33^▪^].

Other diagnostics in development include antibody-in-lymphocyte-supernatant (ALS), which has demonstrated good sensitivity and specificity in endemic settings [34–36]. Several polymerase chain reaction (PCR)-based methods have also been developed and demonstrate promising results in some small-scale studies, but there are currently no widely used and validated assays in general use, and remain poorly sensitive. The sensitivity of PCR-based assays can be improved by incorporating a pre-enrichment step [37,38]. Limited laboratory infrastructure, cost, and the length of time required to obtain results currently serve as deterrents to scalability and expansive deployment for both molecular diagnostics and ALS.

Future directions for diagnostic biomarker discovery include the application of high-throughput technologies on clinical specimens, including mass spectrometry [39], next-generation sequencing, and antigen arrays [40,41]. Using mass spectrometry on serum samples from enteric fever patients, Näsström and colleagues have identified a set of metabolites that was able to distinguish typhoid from paratyphoid fever and enteric fever febrile, typhoid negative controls and chronic carriers [42,43,44^▪^]. Transcriptional data from individuals with acute typhoid fever could also be used to identify signatures reliably identifying enteric fever cases [45,46].

## TREATMENT

The mortality rate of enteric fever in the preantibiotic era was estimated to be between 10 and 30%. The availability of traditional first-line antimicrobials over the past nearly 70 years (chloramphenicol, ampicillin, and trimethoprim-sulfamethoxazole) has reduced the overall mortality rate to less than 1%. Unfortunately, their use has been limited by the emergence of so-called multidrug-resistant (MDR) strains, defined as resistance to all three of the ‘traditional’ first-line antimicrobials. Resistance in MDR strains are typically conferred via IncHI1 plasmids, harboring resistance genes such as *catA*, *sul1*, *sul2*, *dfrA*, *bla*_*TEM-1*_, *strA*, *strB*, *tetA*, *tetB*, *tetC,* and *tetD* on composite transposons. These MDR-associated genes have also been known to integrate within the chromosome of H58 *S*. *Typhi* in isolates from countries including India, Nepal, and Bangladesh [47^▪▪^].

MDR strains were responsible for several outbreaks of enteric fever in the 1980/1990s and led to the widespread use of fluoroquinolones as first-line therapy [2]. Despite considerable success in treatment of MDR typhoid, the extensive use of fluoroquinolones has since led to the emergence of intermediate and fully fluoroquinolone resistant strains. Fluoroquinolone resistance occurs mainly via chromosomal mutations in the *gyrA*, *gyrB*, *parC,* and *parE* genes. Cumulative mutations correspond to the degree of fluoroquinolone, for example, a single nucleotide polymorphism (SNP) in codon S83F of *gyrA* will produce a low-level resistance (ciprofloxacin minimal inhibitory concentration [MIC] of 0.125–0.25 mg/l) whereas additional SNPs in *gyrA* (D87N) and *parC* (S80I) confer a higher level of ciprofloxacin resistance (MIC 8–64 mg/l). In 2017, the World Health Organisation designated fluoroquinolone-resistant *Salmonella* spp. as a high priority pathogen, identified as one of 12 families of bacteria thought to pose the greatest risk to human health through rising antimicrobial resistance (AMR) [48].

Third-generation cephalosporins are commonly used in the empirical treatment of enteric fever and are a valuable empirical treatment option in the setting of MDR and of fluoroquinolone resistant isolates [49]. However, a recent typhoid outbreak in Sindh, Pakistan was attributable to so-called extensively drug resistant (XDR) *S. Typhi* H58 defined as an MDR resistance pattern, combined with fluoroquinolone and cephalosporin resistance [50^▪▪^]. In this outbreak strain, cephalosporin resistance was mediated by the horizontal acquisition of a plasmid encoding the *bla*_CTX-M-15_ extended-spectrum β-lactamase, in addition to quinolone resistance containing genes such as *qnrB2*, *qnrB4*. The emergence of cephalosporin resistance calls for an urgent reappraisal in the use of cephalosporins for treating enteric fever in South and South-East Asia severely limits potential treatment options and emphasizes the importance of disease prevention.

Azithromycin is increasingly used for the empirical treatment of enteric fever. Although currently rare, isolates with increased azithromycin MICs and treatment failure have been reported [51]. Azithromycin resistance is known to be mediated via the *ereA*, *msrD,* and *msrA* genes [47^▪▪^].

The monobactam aztreonam may be a treatment option for treatment of fluoroquinolone-resistant *S. Typhi*, particularly in individuals allergic to penicillin. Alternative treatments include tigecycline or carbapenems, but there are relatively limited studies describing their use for the treatment of typhoid fever and widespread use may be limited by the cost of treatment [52].

Combination antibiotic therapy is sometimes used in the treatment of enteric fever, particularly when response to treatment is slow; susceptibilities are unknown and when the diagnosis is uncertain [53]. Combination therapy may have synergistic effects and reduce the rate of emergence of antibiotic-resistant strains. There is currently limited evidence from randomized trials to guide this approach [54,55].

**FIGURE 1:**
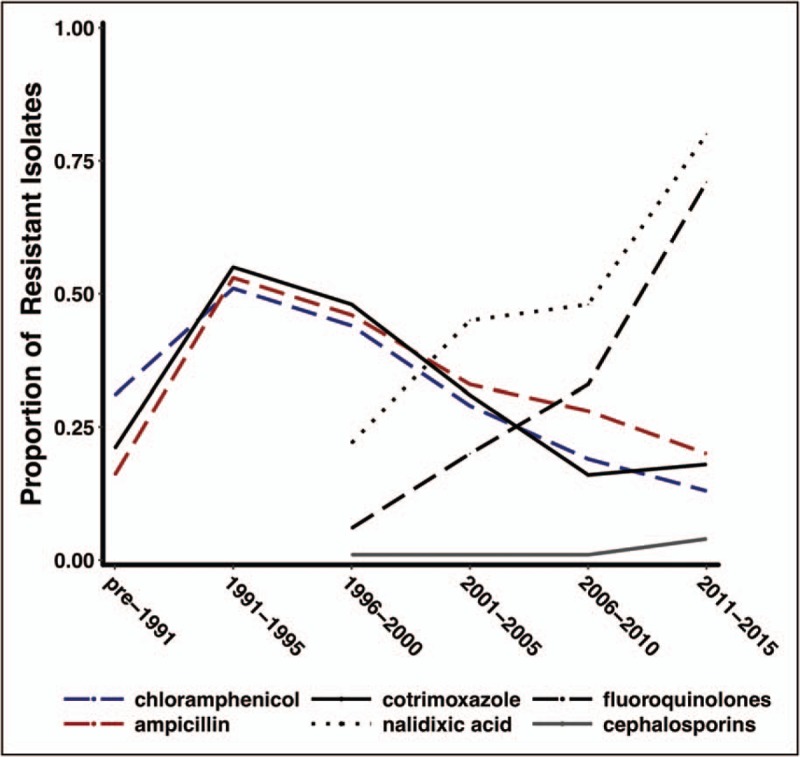
Trends in antimicrobial susceptibility of *S. Typhi* over time. The graph illustrates the proportion of a global collection of *S. Typhi* isolates resistant to different antimicrobial agents, systematically consolidated from published reports from endemic and epidemic sources (1973–2015).

Chloramphenicol, ampicillin, and trimethoprim-sulfamethoxazole were seldom used after widespread MDR strains emerged in the 1990s, but recent data suggest that sensitivity to these agents is re-emerging following their declining use [22^▪^]. One study spanning a 9-year period in Nepal suggesting that over 95% of isolates were sensitive to all three ‘traditional’ first-line antimicrobials [56] – Fig. 1. However, it is almost inevitable that MDR strains will re-emerge over time with the build-up of sufficient antimicrobial pressure via uncoordinated and widespread use of these drugs, particularly if coupled with poor surveillance systems. Nevertheless, these drugs could be used in settings with high fluoroquinolone resistance and evolving cephalosporin resistance – possibly when used in combination or with antibiotic cycling.

Analysis of a global collection of *S*. *Typhi* isolates using whole genome sequencing has demonstrated how a single *S*. *Typhi* haplotype, termed H58 (or genotype 4.3.1.), has emerged and spread globally. Strains of *S*. *Typhi* of H58 are associated with multidrug resistance and reduced fluoroquinolone susceptibility, and isolates belonging to this genotype isolates are widely prevalent is South and South-East Asia, as well as in Central/Southern Africa [47^▪▪^]. Recent analysis suggests that this haplotype is displacing antibiotic-sensitive strains and may possess a fitness advantage compared with other isolates [47^▪▪^,^57^]. In addition, several MDR typhoid epidemics have recently been described in Africa, evolving independently of the H58 haplotype, suggesting that *S. Typhi* is constantly adapting to new antibiotics and distinct ecological niches [58].

### Antimicrobial treatment options

Empirical treatment guidelines for typhoid fever, where available, are frequently outdated and require updating to reflect global trends in AMR. The majority of randomized trials comparing treatments for enteric fever have a small sample size and lack statistical power to detect meaningful differences between interventions, and the optimal choice of antibiotics and duration of therapy are often uncertain [59]. Differences in study end-points, and failure to report outcomes in culture-negative suspected enteric fever, also complicate the interpretation of trial data [49]. A summary of antimicrobial treatment trails is presented in Supplementary Appendix 1.

Recent studies suggest that fluoroquinolones should not be recommended for empirical treatment of enteric fever in South Asia, due to high risk of treatment failure [71]. Current AMR profiles suggest that ceftriaxone or azithromycin represent appropriate empirical treatment options in South and South-East Asia, whereas fluoroquinolones may represent an appropriate treatment in parts of Africa, where high-level fluoroquinolone resistance is currently less common. Oral azithromycin may be more convenient and cost-effective than parenteral ceftriaxone for outpatient management, but widespread use of this drug in many parts of South Asia today may rapidly lead to development of resistance, highlighting the importance of good microbiological surveillance. Some authors have advocated the addition of doxycycline for suspected enteric fever cases in regions of South Asia with high incidence of *Rickettsia* spp [49,60].

Treatment of chronic carriage may require a combination of medical and surgical interventions [2]. Fluoroquinolones are commonly used in the treatment of chronic carriage and treatment with a 28 day of course of ciprofloxacin (750 mg twice daily) or norfloxacin (400 mg twice daily) can achieve clearance in over 80% of patients. Shorter courses (14 days) of fluoroquinolones may also be efficacious in the treatment of chronic carriage, with treatment success ranging from 87 to 100% in published studies. A prolonged treatment course with azithromycin (28 days) may be of use in the management of chronic carriers infected with fluoroquinolone-resistant isolates, although this has not been formally studies in randomised controlled trials. Cholecystectomy may be required in the presence of cholelithiasis, the efficacy of which is likely to be improved by concomitant administration of antibiotics. Patients with concomitant *Schistosoma* infection should receive antiparasitic treatment with praziquantel to manage chronic urinary and intestinal carriage [2,61].

## PREVENTION

The contribution of unsafe drinking water has been recognized as central to the spread of typhoid fever for over 150 years. Access to clean, safe drinking water – combined with investment in sanitation and hygiene interventions – will be key to reducing the global burden of typhoid fever.

The sustained high burden of disease, coupled with the emergence of drug-resistant strains of *S. Typhi*, makes prevention via vaccination a priority in the short-to-medium term. The Ty21a and Vi-polysaccharide vaccines have demonstrated efficacy at 2 years of 58% (95% CI 40–71%) and 59% (95% CI 45–69%), respectively, but have limited use in the youngest age-group of children due to inconvenience in vaccine administration and poor immunogenicity, respectively [62]. School-based campaigns as well as delivery strategies of these vaccines using the available healthcare structure have been effective in terms of coverage and cost-effectiveness in Asia [63].

TCVs, in which Vi-polysaccharide is covalently linked to carrier proteins, offer several potential advantages over earlier generation typhoid vaccines. The appeal of Vi-conjugate vaccines, relates to their capacity to induce immune responses in infants, enhanced immunogenicity in terms of antibody magnitude, quality and duration, and the potential for boosting of immune responses with revaccination. Proof-in-principle of TCV efficacy is derived, primarily, from trials of a prototype Vi-rEPA vaccine, which demonstrated efficacy of up to 91% (95% CI 77–97%) at 2 years, when given as a two dose schedule in 2–5 year-old children [64]. The Vi-rEPA vaccine was efficacious up to at-least 5 years and was compatible with coadministered expanded programme on immunisation vaccines, but has yet to be commercialised.

The most advanced TCV is the Vi-tetanus toxoid conjugate vaccine, TypbarTCV, manufactured by Bharat Biotech (Hyderabad, India). This vaccine is immunogenic and safe in children from as young as 6 months of age, as well as demonstrating superior immunogenicity to a Vi-polysaccharide vaccine [65]. Importantly, TypbarTCV has demonstrated efficacy of between 54.6 and 87.1% in a stringent controlled human infection model, depending on the efficacy endpoint [66^▪▪^]. Modelling studies have estimated a vaccine efficacy for TypbarTCV of 85% based on serological data [67]. Cost-effective models indicate that routine infant TCV vaccination is likely to be cost-effective in medium- or high-incidence settings, depending on the intervention strategy used and at a modest vaccine cost (∼$2/dose) [68^▪^,69^▪^]. Additional safety and immunogenicity data will be generated in an upcoming introduction of TCV in Navi Mumbai, India and from three-phase IV effectiveness studies conducted as part of the TyVAC consortium. Several TCVs are currently in development, including VI-DT, Vi-rEPA, Vi-CRM_197_, and Vi-tetanus toxoid conjugates, many of which have completed Phase 1 and 2/3 trials [65,70,71].

In October 2017, the WHO Strategic Advisory Group of Experts on immunization recommended programmatic use of TCVs in typhoid endemic countries [72^▪▪^]. The recommendations focussed on the use of TCV from the age of 6 months onward, administered as a single dose, and combined with programmatic administration in combination with other childhood vaccines [72^▪▪^]. Where feasible and supported by epidemiologic data, catch-up vaccination up to 15 years of age was also recommended. The position paper highlights that the roll of TCVs should be prioritized in countries with a high burden of typhoid fever or high rates of AMR. TYPBAR-TCV was prequalified by the WHO in January 2018 [73] and Gavi has committed an $85 million funding window to support the roll out of these vaccines in eligible countries between 2019 and 2020.

Momentum to achieve control of typhoid is building, driven by the availability of effective tools and support from key stakeholders. The challenge now facing the community is to support access to typhoid vaccines where they are needed most.

## CONCLUSION

Typhoid and paratyphoid fever are diseases of poverty. Although they have been virtually eradicated in the developed world, enteric fever remains a major public-health problem in resource-limited settings. Several challenges remain, including in the fields of diagnostics, disease epidemiology, and treatment. Ongoing surveillance is required to monitor dynamic antibiotic resistance profiles of *S. Typhi* and *S. Paratyphi*, including the emergence of resistance to cephalosporins, and the apparent re-emergence of strains sensitive to traditional first-line agents. Further studies are required to assess novel treatment strategies, including adjunctive treatments, novel antimicrobials, antibiotic cycling, and combination therapies.

The development and impending roll-out, of cost-effective, scalable TCVs represents a major advance in typhoid control and could have a major impact on the global burden of disease. TCVs are potentially a valuable tool that overcomes some of the limitations of existing typhoid vaccines and – for the first time – offers us a vaccine that is suitable for routine use in childhood immunization programmes. Earlier generation TCVs have proven highly efficacious in field settings, and a WHO prequalified TCV is efficacious in a controlled human infection model. There is now sufficient evidence to support the roll-out of TCVs in the field bolstered by policy, regulatory, and financial support from key stakeholders including WHO and Gavi. Ongoing studies, including those conducted through the TyVAC consortium [20], will study the impact of these vaccines in a diverse range of setting.

Several opportunities for achieving control of typhoid have been missed in the past. Efficacy data for TCVs have been available since 2001, but a number of hurdles have resulted in a failure to build on these promising results. In addition, WHO recommendations for programmatic deployment of Vi-polysaccharide and Ty21a vaccines in 2008 had very limited uptake. In the intervening years, no new diagnostics have been deployed, resistance to antimicrobials has worsened and millions of the people continue to suffer from typhoid every year. In order to learn the lessons of the past, we advocate for the deployment of TCVs in line with WHO recommendations, to improve child health and limit the spread of antibiotic resistance.

## Acknowledgements

The author wish to thank Eli Harris (Healthcare Libraries, Bodleian Library, University of Oxford) for assistance with the literature review.

### Financial support and sponsorship

The authors acknowledge the support of the Wellcome Trust (Strategic award no 106158/zZ/14/Z) and the Bill & Melinda Gates Foundation (no.617OPP1141321) for funding of The Strategic Typhoid alliance across Africa and Asia (STRATAA) which supported this review; the National Institute for Health Research (NIHR) Oxford Biomedical Research Centre and the NIHR Thames Valley and South Midlands Clinical Research Network. C.B. is a Rhodes scholar funded by the Rhodes Trust. A.J.P. is a NIHR Senior Investigator. The views expressed in this article are those of the author(s) and not necessarily those of the NHS, the NIHR, or the Department of Health. M.M.G. and C.B. conducted the literature review and wrote the draft manuscript. A.J.P. supervised the work and edited all subsequent versions of the manuscript.

Declarations of Interest Statement: A.J.P. chairs the UK Department of Health's (DH) Joint Committee on Vaccination and Immunisation (JCVI) and is a member of the WHO's Strategic Advisory Group of Experts on Immunisation. The views expressed in this manuscript are those of the authors and do not necessarily reflect the views of the JCVI, the NHS, the NIHR, UK the Department of Health, or the WHO.

### Conflicts of interest

There are no conflicts of interest.

## REFERENCES AND RECOMMENDED READING

Papers of particular interest, published within the annual period of review, have been highlighted as:

▪ of special interest
▪▪ of outstanding interest

## Supplementary Material

Supplemental Digital Content
