# Supplementary material for: Typhoid and paratyphoid fever: a call to action
Source: Curr Opin Infect Dis. 2018 Aug 17;31(5):440–8. doi: 10.1097/QCO.0000000000000479 (PMC6319573; doi:10.1097/QCO.0000000000000479)
Supplement: Supplemental Digital Content [file coidi-31-440-s001.docx]

# Appendix 1

## Supplementary Table 1 – Summary of trials for antibiotic treatment of enteric fever 1990 to 2017.

| **Author**  **Year**  **Country**  **Reference No.** | **Antimicrobial** | **Sample size** | **Duration** | **Age range** | **Route of**  **administration** | **Summary** |
| --- | --- | --- | --- | --- | --- | --- |
| Lasserre  1991  The Philippines (1) | Ceftriaxone 3g daily  Vs.  Ceftriaxone 4g daily  Vs.  Chloramphenicol 500mg QDS | 19  20  20 | 3 days  3 days  14 days | 15 – 50 years | Parenteral | Clinical cure: Ceftriaxone 3g 94% vs. Ceftriaxone 100% vs. Chloramphenicol 100%  Relapse: Ceftriaxone 3g 2/19 vs. Ceftriaxone 4g 0/20 vs. Chloramphenicol 3/20 |
| Islam  1993  Bangladesh (2) | Ceftriaxone (75mg/kg children, 4g/day adults)  Vs.  Chloramphenicol (60mg/kg/day to defevervesence followed by 40mg/kg to Day 14) | 28  31 | 5 days  14 days | 6 months – 60 years | Parenteral  Oral or Parenteral | Clinical cure: Ceftriaxone 22/28 vs. Chloramphenicol 28/31  Relapse: Ceftriaxone 1/28 vs. Chloramphenicol 1/31 |
| Gotuzzo  1994  Peru  (3) | Aztreonam  (2g IV TDS)  Vs.  Chloramphenicol (50mg/kg QDS) | 22  22 | 10 days  14 days | >14 years | IV  PO/IV | Higher treatment failure with aztreonam compared with chloramphenicol (32% vs. 0%). Shorter fever clearance times with aztreonam. |
| Acharya  1995  Nepal  (4) | Ceftriaxone (50 mg/kg)  vs  Chloramphenicol (60 mg/kg) | 8  8 | 3 days  14 days | Not reported | Parenteral | No difference in duration of clinical cure or onset of complications between the two groups.  Fever clearance time:  Treatment failure: 17% vs. 13% |
| Girgis  1995  Egypt  (5) | Cefixime (20-30 mg/kg) | 60 | 8 days | 3 -16 years | Oral | Cefixime given as an 8 day course is safe and effective in the management of MDR typhoid in children.  Clinical cure: 95% |
| Girgis  1995  Egypt  (6) | Cefixime (7.5 mg/kg)  vs  ceftriaxone (50 – 70 mg/kg)  vs  azetronam ( 50 – 70 mg/kg) | 50  43  43 | 14 days  5 days  7 days | Not reported | Oral  Parenteral  Parenteral | All the 3 regimes were equally safe and efficacious. While ceftriaxone was more economical for in-patient care cefixime was better for out-patient care.  Fever clearance time: Cefixime 5.3 days vs. Ceftriaxone 3.9 days vs. aztreonam 5.5 days  Relapse: Cefixime 6% vs. ceftriaxone 5% vs. aztreonam 6% |
| Butler  1999  India  (7) | Azithromycin (500mg OD)  Vs.  Chloramphenicol (2-3g in four divided doses) | 42  35 | 7  14 | >18 years | Oral  Oral | Azithromycin given once daily for 7 days was equivalent in effectiveness to chloramphenicol when given to patients with chloramphenicol-susceptible infections.  Clinical cure: Azithromycin 88% vs. Chloramphenicol 86% |
| Girgis  1999  Egypt (8) | Azithromycin  (1g on Day1, 500mg OD thereafter)  Vs.  Ciprofloxacin (500mg BD) | 36  28 | 7  7 | >18 years | Oral  Oral | Azithromycin and ciprofloxacin showed equivalent clinically and bacteriologically efficacy against typhoid fever caused by both sensitive organisms and MDRS. Typhi.  Mean fever clearance time: Azithromycin 3.8 days vs. Ciprofloxacin 3.3 days.    No cases of relapse. |
| Cao  1999  Vietnam (9) | Ofloxacin (10 mg/kg)  vs  cefixime (20 mg/kg) | 38  44 | 5 days  7 days | <15 years | Oral  Oral | Higher rate of treatment failures in the cefixime group (11 vs. 1).  Median fever clearance times:  Ofloxacin 4.4 days vs Cefixime 8.5 days |
| Chinh  2000  Vietnam (10) | Azithromycin (1g daily, 20mg/kg/day)  Vs  Ofloxacin (200mg BD, 8mg/kg/day) | 44  44 | 5  5 | ≥15 years | Oral  Oral | A 5-day course of azithromycin was effective for the treatment of enteric fever due to MDR and nalidixic-acid-resistant S. Typhi,  Clinical cure: Azithromycin 95.5% vs. 86.4%, p=ns  Patients with nalidixic acid-resistant typhoid treated with ofloxacin had a longer mean fever clearance time compared with those treated with azithromycin (174hrs vs 135 hrs) |
| Bhutta  2000  Pakistan (11) | Ceftriaxone (65 mg/kg) | 29  28 | 7 days  vs  14 days | 2 – 11 years | Parenteral | 14% of children in the short duration treatment arm had bacteriological relapse within 4 weeks as opposed to 0 % in the long duration arm. |
| Frenck  2000  Egypt (12) | Azithromycin (10 mg/kg)  vs  ceftriaxone (75 mg/kg) | 31  29 | 7 days | 4 -17 years | Oral  Parenteral | Oral azithromycin is safe, effective and convenient for uncomplicated paediatric enteric fever.  Clinical cure by day 7: Azithromycin 91% vs. Ceftriaxone 97%  Mean fever clearance time: Azithromycin 4.1 days vs. Ceftriaxone 3.9 days |
| Gasem  2003  Indonesia (13) | Ciprofloxacin (500mg BD)  vs.  Chloramphenicol (500mg QDS) |  | 7 days  7 days | >14 years | 28  27 | Median fever clearance time: 5.1 days vs. 5.7days  Treatment failure: Chloramphenicol 5/27 vs. Ciprofloxacin 1/28  Relapse: 0% per group (NB follow up to Day 14 only) |
| Tatli  2003  Turkey (14) | Ceftriaxone  (75 mg/kg)  vs  Chloramphenicol (75 mg/kg) | 36  36 | 8 – 12 days  14 days | <16 years | Parenteral  Oral | Clinical cure without complications was achieved in all patients.  Fever clearance: Ceftriaxone 5.4 days vs. chloramphenicol 4.2 days.  Relapse: ceftriaxone 0% vs. 14% chloramphenicol group. |
| Frenck  2004  Egypt (15) | Azithromycin (20 mg/kg)  vs  ceftriaxone (75 mg/kg) | 32  36 | 5 days | 3 -17 years | Oral  Parenteral | A 5-day course of azithromycin was found to be an effective treatment for uncomplicated typhoid fever in children and adolescents.  Clinical cure: azithromycin 94% vs. ceftriaxone 97%  Mean fever clearance time: azithromycin 4.5 days vs. ceftriaxone 3.6 days. |
| Phongmany 2005  Laos (16) | Ofloxacin (15mg/kg)  Vs.  Chloramphenicol (50mg/kg) | 27  23 | 3 days  14 days | >15 years | Oral  Oral | Three days ofloxacin was more effective than 14 days chloramphenicol for the in-patient treatment of typhoid fever, irrespective of antibiotic susceptibility.  Treatment failure: Chloramphenicol 1/23 vs. Oxfloxacin 0/27  Median fever clearance: Ofloxacin 54hrs vs. Chloramphenicol 90 hours. |
| Vinh  2005  Vietnam (17) | Ofloxacin (10 mg/kg) | 89  107 | 2 days  vs  3 days | Not reported | Oral | No significant difference in fever clearance time or duration of hospitalization in children with uncomplicated enteric fever.  Treatment failure: 13.5% vs. 7.5%  Mean fever clearance time: 92hrs vs 101hrs |
| Shakur  2007  Bangladesh (18) | Cefpodoxime proxetil (16 mg/kg)  vs  cefixime (20 mg/kg) | 21  19 | 10 days | 6 months – 12 years | Oral | Clinical efficacy similar in both groups.  Mean fever clearance time 4.87 days vs 4.27 days  1 relapse per group. |
| Parry  2007  Vietnam (19) | azithromycin (10 mg/kg)  vs  ofloxacin (15 mg/kg) + azithromycin (10mg/kg)  vs  Ofloxacin (20 mg/kg) | Not reported | 7 days | Not reported | Oral  Oral  Oral | Fever clearance time:  5.8 days vs. 8.2 days vs. 7.1 days  Treatment failure: 11/62 (18%) vs. 15/62 (24%) vs. 23/63 (37%)  Fever clearance time for patients treated with azithromycin was shorter than that for patients treated with ofloxacin + azithromycin and ofloxacin.  A 7-day course of azithromycin may be used to successfully treat uncomplicated MDR typhoid fever with reduced susceptibility to fluoroquinolones.  No cases of relapse |
| Pandit  2007  Nepal (20) | Gatifloxacin (10 mg/kg)  vs  cefixime (20 mg/kg) | Not reported | 7 days | Not reported | Oral | Gatifloxacin was more efficacious in terms of defeversence time, treatment failure and relapse.  Treatment failure: Gatfloxacin 3.5% vs. Cefixime 37.6%  Median fever clearance time: Gatifloxacin 92 hours vs. Cefixime 138 hours.  Relapse: Gatifloxacin 3.4% vs. cefixime 12.4% |
| Dolecek  2008  Vietnam (21) | Gatifloxacin (10 mg/kg)  vs  azithromycin (20 mg/kg) | 109  101 | 7 days | 2 -15 years | Oral | Both antimicrobials are equally efficacious with gatifloxacin being one-third the cost of azithromycin.  Treatment failure: gatifloxacin 9% vs. azithromycin 9.3%.  Median fever clearance time: gatifloxacin 106 hours vs. azithromycin 106 hours. |
| Arjyal  2011  Nepal (22) | Gatifloxacin (10 mg/kg)  vs  chloramphenicol (75 mg/kg) | Not reported | 7 days  14 days | Not reported | Oral | Gatifloxacin may be the preferred treatment because of its shorter treatment duration and fewer adverse effects.  Treatment failure: Gatifloxacin 6.7% vs. Chloramphenicol 8%  Median fever clearance time: Gatifloxain 3.9 days vs. Chloramphenicol 3.95. |
| Chandney  2012  India (23) | Azithromycin(1g on day 1 500mg/day therafter)  vs  Ofloxacin (20 mg/kg) | 20  20 | 7 days | 18 to 60 years | Oral | Equivalent efficacicacy of azithromycin and ofloxacin in of typhoid fever.    Mean fever clearance time: Ofloxacin 3.68 days vs. Azithromycin 3.65 days |
| Koirala  2013  Nepal (24) | Gatifloxacin (10 mg)  vs  Ofloxacin (20 mg) |  | 7 days | ≥2 years | Oral | Gatifloxacin was not superior to ofloxacin in preventing failure, but use of gatifloxacin did result in more prompt fever clearance time compared to ofloxacin. |
| Arjyal  2016  Nepal (25) | Gatifloxacin (10mg) vs ceftriaxone (60mg) |  | 7 days | 2 -13 years | Oral  Parenteral | Fluoroquinolones should no longer be used for treatment of enteric fever in Nepal* |

# References

1. Lasserre R, Sangalang RP, Santiago L. Three-day treatment of typhoid fever with two different doses of ceftriaxone, compared to 14-day therapy with chloramphenicol: a randomized trial. J Antimicrob Chemother [Internet]. 1991 Nov [cited 2017 Aug 28];28(5):765–72. Available from: http://www.ncbi.nlm.nih.gov/pubmed/1778879

2. Islam A, Butler T, Kabir I, Alam NH. Treatment of typhoid fever with ceftriaxone for 5 days or chloramphenicol for 14 days: a randomized clinical trial. Antimicrob Agents Chemother. 1993 Aug;37(8):1572–5.

3. Gotuzzo E, Echevarria J, Carrillo C, Sanchez J, Grados P, Maguina C, et al. Randomized Comparison of Aztreonam and Chloramphenicol in Treatment of Typhoid Fever. Antimicrob Agents Chemother. 1994;38(3):558–62.

4. Acharya G, Butler T, Ho M, Sharma PR, Tiwari M, Adhikari RK, et al. Treatment of typhoid fever: randomized trial of a three-day course of ceftriaxone versus a fourteen-day course of chloramphenicol. Am J Trop Med Hyg. 1995 Feb;52(2):162–5.

5. Girgis NI, Tribble DR, Sultan Y, Farid Z. Short course chemotherapy with cefixime in children with multidrug-resistant Salmonella typhi Septicaemia. J Trop Pediatr. 1995 Dec;41(6):364–5.

6. Girgis NI, Sultan Y, Hammad O, Farid Z. Comparison of the efficacy, safety and cost of cefixime, ceftriaxone and aztreonam in the treatment of multidrug-resistant Salmonella typhi septicemia in children. Pediatr Infect Dis J. UNITED STATES; 1995 Jul;14(7):603–5.

7. Butler T, Sridhar CB, Daga MK, Pathak K, Pandit RB, Khakhria R, et al. Treatment of typhoid fever with azithromycin versus chloramphenicol in a randomized multicentre trial in India. J Antimicrob Chemother. 1999;44(2):243–50.

8. Girgis NI, Butler T, Frenck RW, Sultan Y, Brown FM, Tribble D, et al. Azithromycin versus ciprofloxacin for treatment of uncomplicated typhoid fever in a randomized trial in Egypt that included patients with multidrug resistance. Antimicrob Agents Chemother. 1999;43(6):1441–4.

9. Cao XT, Kneen R, Nguyen TA, Truong DL, White NJ, Parry CM. A comparative study of ofloxacin and cefixime for treatment of typhoid fever in children. The Dong Nai Pediatric Center Typhoid Study Group. Pediatr Infect Dis J. 1999 Mar;18(3):245–8.

10. Chinh NT, Parry CM, Ly NT, Ha HD, Thong MX, Diep TS, et al. A randomized controlled comparison of azithromycin and ofloxacin for treatment of multidrug-resistant or nalidixic acid-resistant enteric fever. Antimicrob Agents Chemother. 2000 Jul;44(7):1855–9.

11. Bhutta ZA, Khan IA, Shadmani M. Failure of Short-Course Ceftriaxone Chemotherapy for Multidrug-Resistant Typhoid Fever in Children: a Randomized Controlled Trial in Pakistan. Antimicrob Agents Chemother. 2000 Feb;44(2):450–2.

12. Frenck RW, Nakhla I, Sultan Y, Bassily SB, Girgis YF, David J, et al. Azithromycin versus ceftriaxone for the treatment of uncomplicated typhoid fever in children. Clin Infect Dis. 2000;31(5):1134–8.

13. Gasem MH, Keuter M, Dolmans WM V, Van der Ven-Jongekrijg J, Djokomoeljanto R, Van der Meer JWM, et al. Persistence of Salmonellae in Blood and Bone Marrow: Randomized Controlled Trial Comparing Ciprofloxacin and Chloramphenicol Treatments against Enteric Fever. Antimicrob Agents Chemother. 2003;47(5):1727–31.

14. Tatli MM, Aktas G, Kosecik M, Yilmaz A. Treatment of typhoid fever in children with a flexible-duration of ceftriaxone, compared with 14-day treatment with chloramphenicol. Int J Antimicrob Agents. 2003;21(4):350–3.

15. Frenck RW, Mansour A, Nakhla I, Sultan Y, Putnam S, Wierzba T, et al. Short-course azithromycin for the treatment of uncomplicated typhoid fever in children and adolescents. Clin Infect Dis. 2004;38(7):951–7.

16. Phongmany S, Phetsouvanh R, Sisouphone S, Darasavath C, Vongphachane P, Rattanavong O, et al. A randomized comparison of oral chloramphenicol versus ofloxacin in the treatment of uncomplicated typhoid fever in Laos. Trans R Soc Trop Med Hyg. 2005;99(6):451–8.

17. Vinh H, Duong NM, Phuong LT, Truong NT, Bay PVB, Wain J, et al. Comparative trial of short-course ofloxacin for uncomplicated typhoid fever in Vietnamese children. Ann Trop Paediatr. Maney; 2005 Mar;25(1):17–22.

18. Shakur MS, Arzuman SAL, Jesmin H, Hasan M, Mesbahuddin A. Cefpodoxime proxetil compared with cefixime for treatment of typhoid fever in children. Indian Pediatr. 2007;44(11):838–41.

19. Parry CM, Ho VA, Phuong LT, Van Be Bay P, Lanh MN, Tung LT, et al. Randomized controlled comparison of ofloxacin, azithromycin, and an ofloxacin-azithromycin combination for treatment of multidrug-resistant and nalidixic acid-resistant typhoid fever. Antimicrob Agents Chemother. 2006/12/06. 2007;51(3):819–25.

20. Anil P, Amit A, Day JN, Buddhi P, Dangol S, Zimmerman MD, et al. An open randomized comparison of gatifloxacin versus cefixime for the treatment of uncomplicated enteric fever. PLoS One. 2007;(6):e542.

21. Dolecek C, La TTP, Rang NN, Phuong LT, Vinh H, Tuan PQ, et al. A multi-center randomised controlled trial of gatifloxacin versus azithromycin for the treatment of uncomplicated typhoid fever in children and adults in Vietnam. PLoS One. 2008;3(5).

22. Arjyal A, Basnyat B, Koirala S, Karkey A, Dongol S, Agrawaal KK, et al. Gatifloxacin versus chloramphenicol for uncomplicated enteric fever: an open-label, randomised, controlled trial. Lancet Infect Dis. 2011 Jun;11(6):445–54.

23. Chandey M, Multani AS. A comparative study of efficacy and safety of azithromycin and ofloxacin in uncomplicated typhoid fever: A randomised, open labelled study. J Clin Diagnostic Res. 2012;6(10):1736–9.

24. Koirala S, Basnyat B, Arjyal A, Shilpakar O, Shrestha K, Shrestha R, et al. Gatifloxacin Versus Ofloxacin for the Treatment of Uncomplicated Enteric Fever in Nepal: An Open-Label, Randomized, Controlled Trial. PLoS Negl Trop Dis. 2013;7(10).

25. Arjyal A, Basnyat B, Nhan HT, Koirala S, Giri A, Joshi N, et al. Gatifloxacin versus ceftriaxone for uncomplicated enteric fever in Nepal: an open-label, two-centre, randomised controlled trial. Lancet Infect Dis. Elsevier; 2016 Jan;16(5):535–45.
